# Water-Induced Finger Wrinkles Do Not Affect Touch Acuity or Dexterity in Handling Wet Objects

**DOI:** 10.1371/journal.pone.0084949

**Published:** 2014-01-08

**Authors:** Julia Haseleu, Damir Omerbašić, Henning Frenzel, Manfred Gross, Gary R. Lewin

**Affiliations:** 1 Department of Neuroscience, Max Delbrück Center for Molecular Medicine, Berlin-Buch, Germany; 2 Department of Audiology and Phoniatrics, Charité Universitätsmedizin, Berlin, Germany; McMaster University, Canada

## Abstract

Human non-hairy (glabrous) skin of the fingers, palms and soles wrinkles after prolonged exposure to water. Wrinkling is a sympathetic nervous system-dependent process but little is known about the physiology and potential functions of water-induced skin wrinkling. Here we investigated the idea that wrinkling might improve handling of wet objects by measuring the performance of a large cohort of human subjects (n = 40) in a manual dexterity task. We also tested the idea that skin wrinkling has an impact on tactile acuity or vibrotactile sensation using two independent sensory tasks. We found that skin wrinkling did not improve dexterity in handling wet objects nor did it affect any aspect of touch sensitivity measured. Thus water-induced wrinkling appears to have no significant impact on tactile driven performance or dexterity in handling wet or dry objects.

## Introduction

Water-induced wrinkling of human non-hairy (glabrous) skin of the fingers, palms and soles is an as yet not fully understood phenomenon influenced by water temperature, pH, and tonicity [1], [2]. Interestingly, glabrous skin that lacks sweat glands, like the clitoris and glans penis, does not wrinkle after water immersion [3]. In the 1930s, Lewis and Pickering first described the absence of wrinkling in patients with median nerve palsy which suggested that the nervous system plays a central role in wrinkling [4]. Since then, other studies have described the dependence of wrinkling on the sympathetic nervous system. These discoveries led to the implementation of the wrinkling test as a bedside test of sympathetic nerve function [5]–[13].

Like water-induced wrinkling, heat-induced vasoconstriction is controlled by the sympathetic nervous system and was shown to occur upon warm water immersion of the glabrous skin which is rich in arterio-venous shunts or anastomoses and sweat glands [14]–[16]. By measuring changes in blood flow in the digital arteries upon water immersion of the hands, Wilder-Smith and Chow [17] showed that water-induced skin wrinkling is directly linked to vasoconstriction. Considering the unique features of the glabrous skin at the extremities, i.e. finger and toe digits, they suggested that dyselectrolytemia caused by water entry through sweat ducts induces sympathetic nervous system-dependent vasoconstriction. The resulting negative pressure in the finger pulp exerts forces on the overlying epidermal layers, which eventually leads to skin wrinkling [18]. Hsieh et al. [11] provided supportive evidence for a causal relationship between water-induced wrinkling and vasoconstriction by measuring blood flow velocity before and after water-immersion of the hands in patients who underwent digital replantation. They observed that upon water immersion the skin of replanted fingers failed to wrinkle and the blood flow was increased (vasodilatatory effect).

Given that water-induced skin wrinkling is controlled by the sympathetic nervous system, Changizi et al. [19] hypothesized that wrinkles may serve an adaptive function in wet conditions. By analyzing the wrinkle pattern of 28 fingers of 13 hands, found in the public online domain, and comparing them to convex mountain promontories they suggested that wrinkles serve as drainage networks for channeling water away during grip in wet conditions (‘rain tread’ hypothesis). A recently published study by Kareklas et al. [20] provided support for the “rain tread” hypothesis with evidence that water-induced wrinkles selectively improve handling of wet objects. In a behavioral study with 20 participants, they showed that subjects transferred submerged objects faster with wrinkled fingers than with non-wrinkled fingers. Furthermore, the authors postulated that, despite not having a detrimental effect on handling dry objects, wrinkles might be disadvantageous in other ways under dry conditions, e.g. by impairing touch sensitivity. We decided to test the above speculation directly by repeating the wrinkling paradigm introduced by Kareklas and colleagues [20] and measuring the effect of wrinkling on measures of touch acuity. In order to perform such a study we chose to reinvestigate the effect of water-induced wrinkling on the handling of wet objects in our human test subjects. We found that touch sensitivity, assessed by measuring tactile spatial acuity with a grid test or vibration detection thresholds at 10 Hz and 125 Hz, was unaffected by wrinkling the finger pad skin. Furthermore, the finding that fingertip wrinkles improve handling of wet objects could not be reproduced in our human cohort.

## Methods

### Ethics Statement

All experiments performed were approved by the local ethics committee (Charité - Universitätsmedizin Berlin, Germany). Each participant was asked to sign a written statement of informed consent. Participants were familiarized with the experimental setups, but were not informed about the specific hypothesis to be tested.

All tests described below (tactile acuity, vibration detection threshold and manual dexterity) were performed on non-wrinkled and wrinkled fingers using a counter-balanced design. Wrinkling was achieved by immersing both hands in 10 L of 40°C water for 30 min. Participants who performed the assigned tasks with wrinkled fingers first were then asked to wash their hands, dry them, and wait for 30 min for the wrinkles to disappear. To guarantee a high degree of skin wrinkling, the tests were run in three sessions: tactile acuity and manual dexterity were tested in one session, vibration detection thresholds at 10 Hz and 125 Hz were determined each in a separate session. After the completion of each test, participants' fingers were visually inspected to confirm the presence of wrinkles.

### Tactile Acuity Test

Tactile acuity was determined with a grating orientation determination test using the Tactile Acuity Cube (grating widths: 0.75–6 mm) as described previously [21]. Briefly, 38 blindfolded participants (age range: 20–35 years, mean age: 27.5±3.3 years; 14 males, 24 females) placed their hand on a table with the palmar surface facing up. The Tactile Acuity Cube was applied for 1 s to the pad of the right index finger (regardless of participants' handedness) in a way that the cube exerted its whole weight on the finger (233 g). Participants had to determine the orientation of the gratings on the cube (parallel or perpendicular to the fingers; the orientation of the gratings was chosen randomly by the experimenter) starting with the widest grating width (6 mm). A 1-up 2-down staircase procedure was used to determine the detection threshold of 70.7% correct [22]. If the orientation was correctly identified twice, the next smallest grating width was tested. This was continued until the participant answered incorrectly. The grating width was then increased again until the grid orientation of a certain width was determined correctly twice in succession. Thirteen of these reversal points were determined and the median of the last 10 were taken as the threshold.

### Vibration Detection Threshold

The vibration detection threshold was determined using a custom designed system configured for a two interval forced choice test design. A piezo actuator (P-801, Physik Instrumente, Karlsruhe, Germany) was mounted to a balanced brass bar so that a weight of 30 g was applied to the skin area being tested (Fig. S1). Despite the relatively low application pressure, the brass bar had a considerable mass (15.5 kg) in order to avoid transmission of the oscillation to the device and minimize dissipation of the sine wave vibration. The piezo actuator, a patient cue display and a response unit were controlled by a PowerLab 4/35 data acquisition system (ADInstruments, Bella Vista, Australia). The probe, a plastic disc of 1 cm diameter, was positioned on the subjects' right index finger pad. Two frequencies (10 and 125 Hz) were tested in separate blocks. A vibration stimulus (stimulus durations were 2.5 and 1.5 s for 10 and 125 Hz, respectively) was applied in one of the two intervals that were indicated visually to the test subjects as “1” and “2” on a screen. The vibration stimulus was randomly (computer generated) applied either during the first (“1”) or the second (“2”) half of each trial. After every trial the participants had to press either of two buttons (“1” or “2”) accordingly. The test was started with a moderate amplitude identifiable by the vast majority of subjects (16.158 µm for both 10 and 125 Hz frequencies). Vibration at a given amplitude was rated “felt” when the test subject identified the correct interval 6 out of 7 times, provided 5 of the first 6 responses were correct. Otherwise the protocol was extended to 9 trials and the respective amplitude was considered “felt” when the test subject identified the intervals 7 out of 9 times correctly. The number of consecutive trials at each amplitude was thus limited to a maximum of 9 in order to estimate a percent correct value of 75.2% [23]. If the vibration at that amplitude was rated “felt”, the next lower amplitude (lowered by a factor of 0.79) was assessed. If the vibration was rated “not felt”, the amplitude was increased by a factor of 1.26. Eight of these reversal points were determined and the median of the last six taken as the threshold. The statistical analyses of the vibration detection thresholds were performed on log10-transformed data to account for the skewness of the data. The vibration test was performed on a cohort of 20 participants (age range: 20–30 years, age mean: 27.9±2.3; 8 males, 12 females). The order of the frequencies tested was counter-balanced across wrinkling status (wrinkled, non-wrinkled) and subjects.

### Manual dexterity test

Forty subjects (age range: 22–35 years, age mean: 27.8±3.0 years; 15 males, 25 females) were split into two groups: Group 1 (age range: 22–35 years, age mean: 26.7±3.6 years; 10 males, 10 females) and Group 2 (age range: 26–33, age mean: 28.9±1.8 years; 5 males, 15 females).

Every participant was asked to manipulate dry and submerged objects with and without wrinkled fingers in a counter-balanced, within-subject 2×2 factorial design exactly as described by Kareklas et al. [20]. Briefly, the time it took participants to transfer 52 objects from a source container into a target container with a 5×5 cm opening was measured. The objects were 32 glass marbles of 16 mm diameter, 2 glass marbles of 23 mm diameter, 4 rubber balls of 26 mm diameter, 12 plastic dice with a side length of 16 mm, 2 brass weights of 200 g and 500 g. Objects were transferred from the right to the left hand, regardless of participants' handedness, using only the thumbs and index fingers by passing the objects through a 5×5 cm hole (height of the transfer hole above the objects: 75 cm (Group 1) and 45 cm (Group 2)). The source container was either filled with water (3.5 L; water height: 12 cm) or was dry.

### Participants

A power analysis of the original data by Kareklas et al. [20] revealed that a cohort size of 9 participants should be sufficient to detect the effect of skin wrinkling on handling wet objects. The sample size estimation assumptions were: power  = 80%, p = 0.05, two-tailed paired t-test, SD of the difference values (wrinkled/wet vs. non-wrinkled/wet) = 13.4, difference (wrinkled/wet vs. non-wrinkled/wet) = 15.1.

In this study, a total of 42 participants were recruited. Forty participants (mostly undergraduate and graduate students) performed the manual dexterity test (20 participants in each Group 1 and Group 2). Thirty-eight participants performed the tactile acuity test (20 participants from Group 1, 17 participants from Group 2 and one additional participant). Twenty participants performed the vibration detection threshold test (4 participants from Group 1, 14 participants from Group 2 and 2 additional participants).

### Statistical Analysis

All the raw data are available as Dataset S1.

Statistical analysis was performed using SPSS (IBM Corp., Armonk, NY, USA), GraphPad Prism (GraphPad, San Diego, CA, USA) and G*Power 3.1.7 [24] statistics programs. The manual dexterity test data were analyzed using a repeated measures analysis of variance (ANOVA) with two within-subject variables (wrinkling status: wrinkled, non-wrinkled; object status: dry, submerged) and one between-subject variable (task order resulting from counter-balancing, i.e. order in which tasks were performed). The effect of wrinkling on psychophysical parameters was examined using a two-tailed paired t-test.

All values are reported as mean ±SEM. Participants' ages are reported as mean ±SD.

## Results

### Psychophysics

Touch sensitivity, assessed by measuring grating orientation threshold (tactile acuity) and vibration detection thresholds on the right index finger pad, was not affected by water-induced wrinkling.

**Figure 1:**
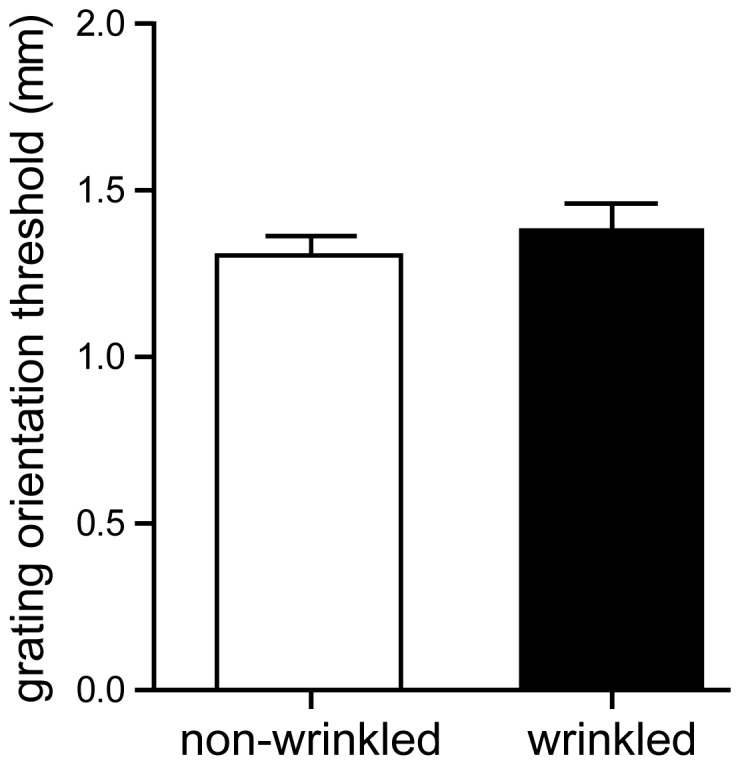
Tactile acuity measured on wrinkled and non-wrinkled finger pads. Wrinkling of the index finger pad skin has no effect on tactile acuity (p = 0.323). Discrimination thresholds (tactile acuity): 1.31±0.06 mm (non-wrinkled fingers, white bar) and 1.39±0.08 mm (wrinkled fingers, black bar). n = 38.

Participants' discrimination thresholds (tactile acuity) were not changed when tested on wrinkled compared to non-wrinkled fingers (1.39±0.08 mm and 1.31±0.06 mm, respectively; two-tailed paired t-test, t(1,37) = 1.001, p = 0.323; Fig. 1).

**Figure 2:**
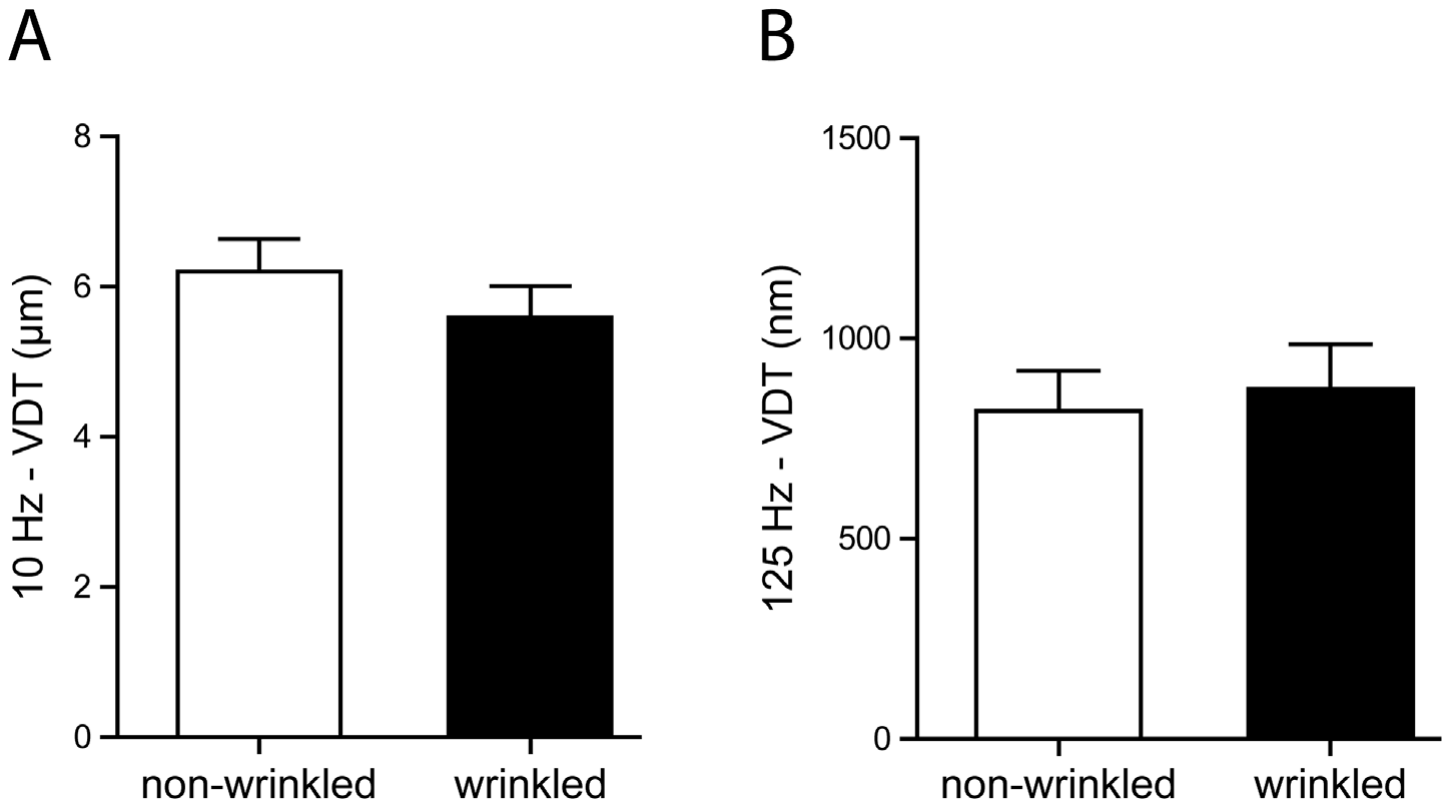
Vibration detection thresholds measured on wrinkled and non-wrinkled finger pads. Wrinkling of the index finger pad skin does not alter vibration detection thresholds (VDT) for 10 Hz and 125 Hz frequencies (two-tailed paired t-test on log10-transformed data, p = 0.133 and p = 0.686, respectively). (**A**) 10 Hz frequency vibration thresholds: 6.19±0.45 µm (non-wrinkled fingers, white bar) and 5.57±0.44 µm (wrinkled fingers, black bar). (**B**) 125 Hz frequency vibration thresholds: 817±102 nm (non-wrinkled fingers, white bar) and 872±114 nm (wrinkled fingers, black bar). n = 20 each.

Furthermore, vibration detection thresholds at 10 Hz and 125 Hz frequencies were not altered by wrinkling of the finger pad skin (two-tailed paired t-test on log10-transformed data, t(1,19) = 1.571, p = 0.133 and t(1,19) = 0.411, p = 0.686, respectively). The average threshold for 10 Hz frequency vibrations was 6.19±0.45 µm in non-wrinkled and 5.57±0.44 µm in wrinkled conditions. At 125 Hz frequency vibrations, the detection thresholds were 817±102 nm when determined for non-wrinkled fingers and 872±114 nm for wrinkled fingers (Fig. 2).

### Manual dexterity test

We measured the time it took participants to transfer dry and submerged objects with and without wrinkled fingers in a 2×2 design as follows: wrinkling status (wrinkled, non-wrinkled) versus object status (dry, submerged). The results were analyzed using a within-subject analysis of variance (ANOVA).

**Figure 3:**
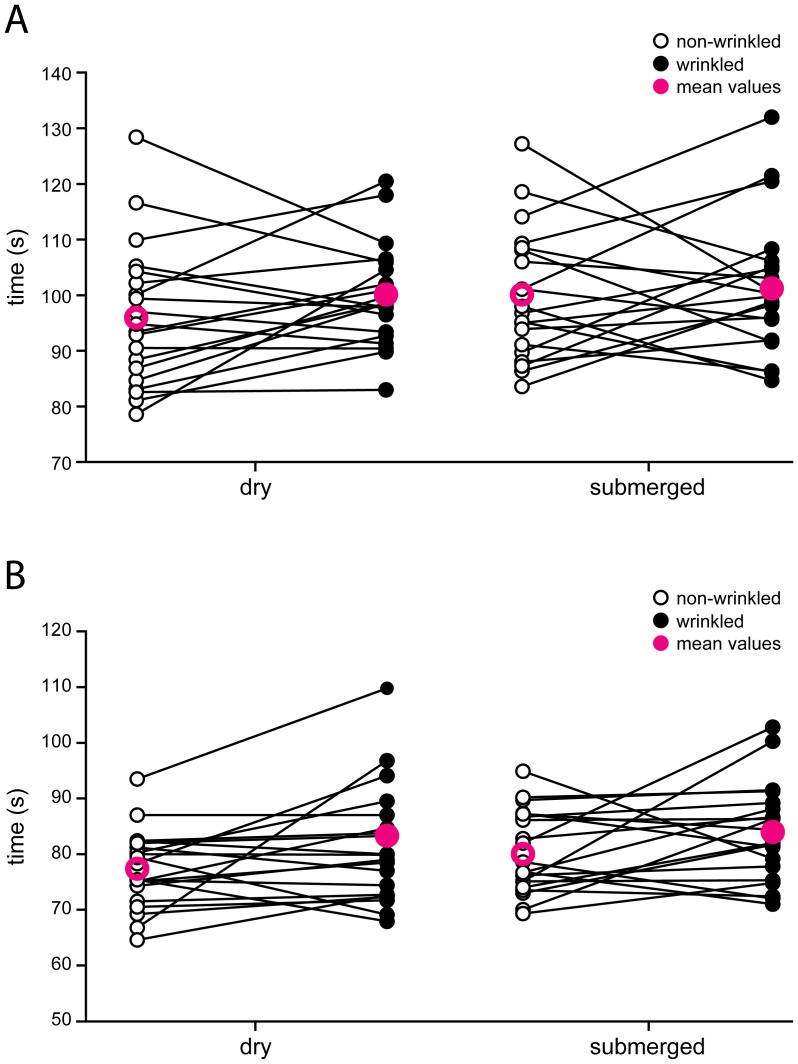
The effect of wrinkling on handling dry and submerged objects. The graphs show the times it took participants to transfer objects from a source container into a target container using only the thumbs and index fingers by passing them through a 5×5 cm transfer hole. (**A**) In Group 1 (n = 20; height of the transfer hole: 75 cm), there were no differences in handling times for dry or submerged objects with wrinkled (filled circles) and non-wrinkled fingers (empty circles) (wrinkling status: F(1, 16) = 2.572, p = 0.128; object status: F(1, 16) = 3.577, p = 0.077; interaction: F(1, 16) = 0.785, p = 0.389; mean values in pink). (**B**) In Group 2 (n = 20; height of the transfer hole: 45 cm), no advantageous effect of having wrinkled fingers on handling submerged objects was observed (object status: F(1, 16) = 3.491, p = 0.080). However, participants were slower in handling both dry and submerged objects with wrinkled than with non-wrinkled fingers (wrinkling status: F(1, 16) = 6.476, p = 0.022). Interaction: F(1,16) = 0.076, p = 0.786.

In Group 1 (height of the transfer hole: 75 cm), neither of the two main effects (wrinkling status: F(1, 16) = 2.572, p = 0.128; object status: F(1, 16) = 3.577, p = 0.077) were statistically significant. In addition, there was no interaction between wrinkling status and object status (F(1, 16) = 0.785, p = 0.389). On average participants transferred dry as well as submerged objects at equal speedwith and without wrinkles (Fig. 3A, Table 1).

**Table 1:** Summary of the times taken to transfer dry and submerged objects with either wrinkled or non-wrinkled fingers (Group 1).

	Wrinkling status		
Object status	Non-wrinkled	Wrinkled	Marginal means (object status)
Dry	96.0±2.8 s	99.9±2.1 s	97.9±2.5 s
Submerged	100.0±2.6 s	101.6±2.7 s	100.8±2.6 s
Marginal means (wrinkling status)	98.0±2.7 s	100.7±2.4 s	

In Group 1 (height of the transfer hole: 75 cm), participants transferred dry as well as submerged objects on average equally rapidly with and without wrinkles.

In the second group (Group 2; height of the transfer hole: 45 cm), analysis of the results revealed one statistically significant main effect. Here the participants were slower in transferring objects with wrinkled fingers compared to non-wrinkled fingers (F(1, 16) = 6.476, p = 0.022). However, as in Group 1, there was no significant effect on performance when participants handled dry or wet objects (F(1, 16) = 3.491, p = 0.080), nor was there a significant interaction effect (F(1,16) = 0.076, p = 0.786). In other words, the effect of the wrinkling status on the time it took participants to transfer dry and submerged objects was the same (Fig. 3B, Table 2).

**Table 2:** Summary of the times taken to transfer dry and submerged objects with either wrinkled or non-wrinkled fingers (Group 2).

	Wrinkling status		
Object status	Non-wrinkled	Wrinkled	Marginal means (object status)
Dry	77.3±1.5 s	81.3±2.3 s	79.3±2.0 s
Submerged	80.4±1.7 s	83.8±2.0 s	82.1±1.8 s
Marginal means (wrinkling status)	78.8±1.6 s	82.6±2.2 s	

In Group 2 (height of the transfer hole: 45 cm), participants transferred dry or wet objects equally rapidly but were slower in transferring objects with wrinkled fingers compared to non-wrinkled fingers.

To account for/cancel out any carryover effects like learning and fatigue we included task order resulting from counter-balancing (i.e. order in which tasks were performed) as a between-subject variable in the analysis. For both Group 1 and Group 2 no effect of the task order was found to be statistically significant (Group 1: F(1, 16) = 0.467, p = 0.709; Group 2: F(1, 16) = 0.381, p = 0.768). Thus, counter-balancing was effective and asymmetric skill transfer could be excluded [25].

## Discussion

It has recently been shown in a small cohort of human volunteers that wrinkled index finger and thumb skin improves performance in a manual dexterity task selectively for handling wet objects as opposed to dry objects [20]. This interesting effect could have been due to an increased tactile sensitivity of the wrinkled glabrous skin providing an advantage for subjects handling wet objects. Here we directly tested this idea by comparing two measures of touch sensitivity on the wrinkled and non-wrinkled skin areas used for manipulating the objects in the task.

We found no evidence that tactile acuity (based on the ability to discriminate the orientation of gratings applied to the fingertip) and vibration detection thresholds were different between wrinkled and non-wrinkled skin. The performance in these two sensory tasks is likely to be dependent on distinct sets of cutaneous mechanoreceptors. Thus spatial acuity in determining grid orientation probably depends predominantly on slowly-adapting type I mechanoreceptors (SAI) that contact Merkel's cells as well as type I rapidly adapting mechanoreceptors (RAI) that terminate in Meissner's corpuscles [26], [27]. In our vibration detection test we used two frequencies that selectively activate RAI mechanoreceptors (10 Hz) or type II RA mechanoreceptors that have Pacinian corpuscle end-organs (125 Hz) [28]–[30]. It is conceivable that the ridges produced by skin wrinkling may alter the visco-elastic properties of the skin and thus the transfer of mechanical force to the receptor endings of Meissner's or Pacinian corpuscles. Some mechanoreceptor end-organs, like Merkel's disks or Meissner's corpuscles are found at superficial locations at the epidermal-dermal interface, and others like Pacinian corpuscles are present deep in the dermis [28]. However, despite changes in the physical nature of the skin induced by wrinkling, we observed no alteration in the psychophysical performance of individuals who were equally capable of detecting vibration at both frequencies whether the skin was wrinkled or not. We conclude that the physiological and physical changes induced by skin wrinkling have either no or negligible impact on the mechanoreceptors that underlie tactile acuity and vibration sensitivity. These findings raised the question of why it is that performance in handling wet objects improves with wrinkled fingers. As part of our study we used the same manual dexterity task devised by Kareklas and colleagues [20] on our own cohorts of human volunteers. We tested two cohorts each with 20 participants (Group 1 and Group 2). Group 1 was tested in a setup where the transfer hole was positioned at a height of 75 cm above the objects. Using this experimental setup, we were not able to reproduce the results presented by Kareklas et al. [20]. Our Group 1 participants handled dry and submerged objects equally fast with wrinkled and non-wrinkled fingers (Fig. 3A).

We decided to repeat the dexterity task with a second cohort of 20 participants (Group 2) in a setup where the transfer hole was lowered to 45 cm above the objects. The rationale for this change was to minimize the complex motor movements of the arms and shoulders. Therefore, the proportion of the actual time needed to grip and manipulate the wet or dry objects was increased. In this situation, participants transferred objects on average faster than with the apparatus used for Group 1 and the between-subject variability was decreased compared to Group 1 participants (Tables 1 and 2). No advantageous effect of wrinkling on handling wet objects was observed in either Group 1 or Group 2. While Group 2 participants were able to transfer dry and wet objects with equal speed, they were slower when transferring objects with wrinkled fingers than with non-wrinkled fingers. It should be noted that the objects used in this study were slightly different in material and number from the ones used in the original study [20]: 52 objects made of glass (34), rubber (4), plastic (12) and brass (2) versus 45 objects made of glass (39) and lead (6). However, we believe that the outcome of the dexterity task should be independent of slight changes in the complexity of the objects, given the hypothesis that finger wrinkles improve handling of wet objects.We conclude that the experimental setup devised by Kareklas et al. [20] may not be suitable to test the hypothesis that wrinkling improves handling of wet objects as suggested by the ‘rain tread’ hypothesis. The fact that we could not reproduce the results presented by Kareklas et al. [20] suggests that this manual dexterity test is not sensitive enough to tease out specific effects of wrinkled skin on performance. The speed of task completion clearly depends on many factors including motivation and motor skills that are independent of the ability to reliably grip objects. Measuring the force required to pull out dry and wet objects held between non-wrinkled or wrinkled fingers would potentially be more suitable to rigorously test the so called ‘rain tread’ hypothesis.

Our evidence does not support the hypothesis that wrinkling of the glabrous skin evolved as an adaptive process to improve handling of wet objects or walking in wet conditions. We suggest that wrinkling does not serve any adaptive function but rather is a byproduct of sympathetic nervous system-induced vasoconstriction that happens upon warm water-immersion. Heat-induced vasoconstriction (HIVC) resulting from immersion of hands and feet in warm water could be a protective mechanism [14] comparable to cold-induced vasodilatation (CIVD) which is thought to prevent cold injuries by locally increasing the tissue temperature (reviewed by Daanen, [31]). In conditions where sweating does not lead to evaporative cooling, heat-induced vasoconstriction could result in a decrease in local heat gain, which is currently controversial [14], [17]. The function of water-immersion induced vasoconstriction triggered by dyselectrolytemia still remains to be clarified.

In summary, the present study shows that glabrous skin wrinkling does not significantly influence touch performance nor does it improve dexterity in handling wet objects.

## Supporting Information

Dataset S1 **Raw data.**
(XLS)

Figure S1 **Vibration stimulator.** To test for vibration detection thresholds, vibration stimuli (10 and 125 Hz) were applied with a pressure of 30 g to the right index finger pad. The threshold amplitudes were determined using a two interval forced choice protocol.
(TIF)
