# Supplementary figures and images for: Water-Induced Finger Wrinkles Do Not Affect Touch Acuity or Dexterity in Handling Wet Objects
Source: PLoS One. 2014 Jan 8;9(1):e84949. doi: 10.1371/journal.pone.0084949 (PMC3885627; doi:10.1371/journal.pone.0084949)

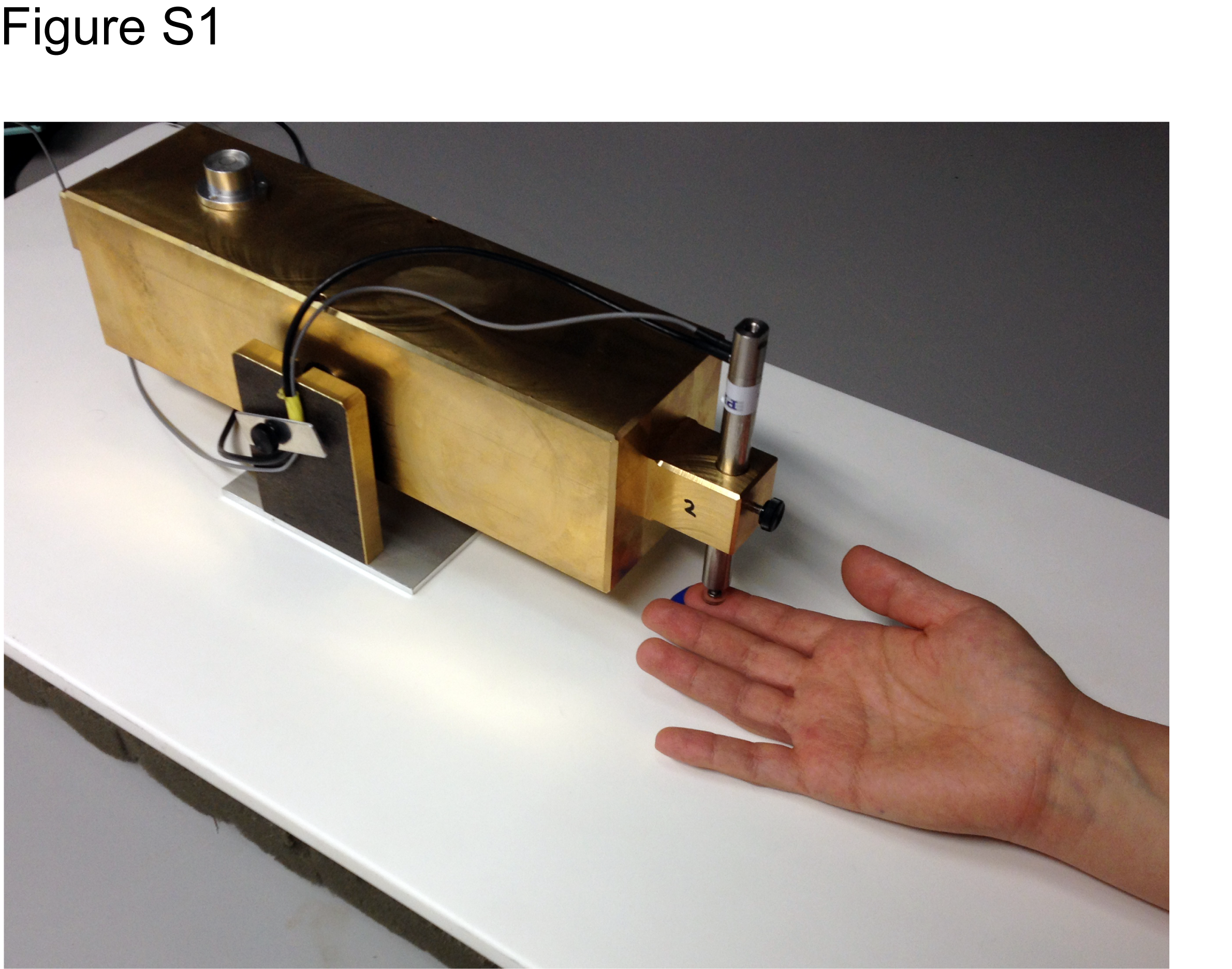

Supplement: Figure S1 — Vibration stimulator. To test for vibration detection thresholds, vibration stimuli (10 and 125 Hz) were applied with a pressure of 30 g to the right index finger pad. The threshold amplitudes were determined using a two interval forced choice protocol. (TIF) [file pone.0084949.s002.tif]
